# Expressional Localization and Functionally Identifying an RNA Editing Enzyme BmADARa of the Silkworm *Bombyx mori*

**DOI:** 10.3390/insects11080523

**Published:** 2020-08-12

**Authors:** Chongjun Ye, Song Jiang, Meixia Gong, Qin Min, Manli Fan, Junshan Gao, Yan Meng

**Affiliations:** 1 School of Life Sciences, Anhui Agricultural University, 130 West Changjiang Road, Hefei 230036, China; yechj1983@163.com (C.Y.); jiangsong2111@126.com (S.J.); 15736938961@163.com (Q.M.); 13625581923@163.com (M.F.); 2 Institute of Sericulture, Anhui Academy of Agricultural Sciences, 15 Huoshan Road, Hefei 230061, China; 3 Anhui International Joint Research and Development Center of Sericulture Resources Utilization, Hefei 230036, China; 4 Guangxi Zhuang Autonomous Region Research Academy of Sericultural Science, 10 Xiajun Road, Nanning 530007, China; gongmx2020@163.com

**Keywords:** *Bombyx mori*, adenosine deaminase, *synaptotagmin I*, A-to-I RNA editing, subcellular localization

## Abstract

**Simple Summary:**

Adenosine deaminase acting on RNA (ADAR) is a key enzyme in the editing of adenosine into inosine (A-to-I). The loss or dysfunction of ADAR enzymes in higher eukaryotes affects the editing efficiency of target genes, leading to some neurological diseases. The silkworm *Bombyx mori* is an oligophagous economically important insect and has been used as an important lepidoptera model insect. By far, the knowledge about A-to-I RNA editing and ADAR members in *B. mori* (BmADAR) is very limited. In this paper, we present a first molecular comprehensive cloning, sequence analysis of BmADAR transcripts and subcelluar localization of BmADARa. As a result, we obtained six BmADAR transcripts encoding different amino and carboxyl termini, among which *BmADARa* is a mainly expressed transcript with complete open reading frame. Our further investigations showed that the majority of BmADARa protein exists in the nucleus and has editing function to a specific site of the silkworm *synaptotagmin I* gene. Overall, by molecular cloning and functional identifing, this paper introduces the first ADAR enzyme in *B. mori* and contributes to further exploration of the functional domain of BmADARa and its editing substrates and target sites.

**Abstract:**

The most common type of RNA editing in metazoans is the deamination of adenosine into inosine (A-to-I) catalyzed by the adenosine deaminase acting on the RNA (ADAR) family of proteins. The deletion or dysfunction of ADAR enzymes in higher eukaryotes can affect the efficiency of substrate editing and cause neurological disorders. However, the information concerning A-to-I RNA editing and ADAR members in the silkworm, *Bombyx mori* (BmADAR), is limited. In this study, a first molecular comprehensive cloning and sequence analysis of *BmADAR* transcripts was presented. A complete open reading frame (ORF) (*BmADARa*) was obtained using RT-PCR and RACE and its expression pattern, subcellular localization and A-to-I RNA-editing function on the silkworm *synaptotagmin I* (*BmSyt I*) were investigated. Subcellular localization analysis observed that *BmADARa* was mainly localized in the nucleus. To further study the A-to-I RNA-editing function of *BmADARa*, BmSyt I-pIZ-EGFP was constructed and co-transfected with BmADARa-pIZ-EGFP into BmN cells. The result demonstrates that BmADARa can functionally edit the specific site of *BmSyt I.* Taken together, this study not only provides insight into the function of the first ADAR enzyme in *B. mori*, but also lays foundations for further exploration of the functional domain of BmADARa and its editing substrates and target sites.

## 1. Introduction

As an RNA-editing enzyme involved in the nucleotide conversion from adenosine to inosine in double-stranded RNA (dsRNA) substrates, adenosine deaminase that acts on RNA (ADAR) was first reported and identified in *Xenopus laevis* [1] and has now been cloned and characterized in many metazoans, including mammals, birds, fish, flies and worms [2,3]. Because the properties of inosine mimic those of guanosine (inosine will form two hydrogen bonds with cytosine, for example), inosine is recognized as guanosine by the translational cellular machinery. Hence, adenosine to inosine (A-to-I) RNA editing has the capacity to diversify gene expression by the alteration of protein sequences, splicing patterns and base-pairing properties [4,5], which is an important mechanism for the occurrence of protein molecular diversity [6]. ADAR family members, ADAR1, ADAR2, ADAR3 and testis nuclear RNA-binding protein (TENR), share some common structures including variable numbers of double-stranded RNA binding motif (dsRBM) in the N-terminal [7], and a highly conserved tRNA-specific and dsRNA adenosine deaminase domain in the C-terminal, and they contain several subcellular localization signals that allow them to shuttle between different compartments, such as the nucleoli, nucleoplasm and cytoplasm [8].

The transcript of the neurotransmitter receptor is an important coding substrate for ADAR. A-to-I editing plays an important role in the nervous system [9]. Recent advances in deep sequencing have enabled the identification of new RNA-editing sites in several organisms. For example, in *Drosophila*, 972 new editing sites in 561 genes were identified, most of which involve neurotransmission [10]. The synaptotagmin gene regulates the transmission of neural signals in the lepidopteran and dipteran, and is also the target gene for ADAR editing [11].

The silkworm *Bombyx mori* is an oligophagous economically important insect that feeds on the mulberry leaf and that has been domesticated for more than 5000 years. *B. mori* has been used as a model for studying insect molecular genetics with complete genome sequencing accomplished after *Drosophila* and *Anopheles* [12]. However, current reports on the interaction of ADAR with substrate RNA, structural and functional identification, and subcellular localization have focused on the research into human nervous system diseases, and there are only a few reports for *Drosophila* [13]. In *B. mori*, several genes with A-to-I RNA-editing sites have been identified. For example, *Dα6* encoding the nicotinic acetylcholine receptor (nAChR) has seven A-to-I RNA-editing sites [14], and *BmKv^2+^* and *BmSynaptotagmin I* (*BmSyt I*) [15] genes have three and two A-to-I RNA-editing sites, respectively. It remains to be seen how the ADAR of *B. mori* (*BmADAR*) selects the target gene and performs A-to-I RNA editing. The editing function in the RNA-editing process also lacks systematic research.

In this study, we used RT-PCR and RACE to clone multiple transcription types of BmADAR genes in the silkworm *B. mori*. The transcript of *BmADARa* with complete ORF sequence was successfully obtained, and the spatiotemporal expression pattern and subcellular localization of BmADARa were studied. We also examined the A-to-I RNA-editing function of BmADARa on *BmSyt I* in order to further explore the functional domain of BmADARa and its mediated A-to-I RNA-editing substrate and target.

## 2. Materials and Methods

### 2.1. Silkworms, Vectors and Cell Strains

The silkworm larvae of Dazao strain were fed fresh mulberry leaves and maintained at 25 °C [16]. The pMD19T plasmid was bought from TaKaRa (Dalian, China). pFastBac^TM^ Dual was always reserved in our laboratory. The pET24b was purchased from Invitrogen. *E. coli* strains DH5α and Transetta (DE3) were purchased from Promega (Madison, WI, USA). pIZ/V5-His-EGFP insect expression plasmid was donated by Prof. Huawei He (Southwest University, China). The ovary-derived cell line BmN of *Bombyx mori* was stored in TC-100 insect culture medium containing 10% fetal bovine serum (ExCell, China) and 1% penicillin-streptomycin (Hyclone, USA) at 27 °C. Sf9 cell lines was obtained from the American Type Culture Collection (ATCC) and maintained in complete TNM-FH insect medium (Sigma-Aldrich, St. Louis, MO, USA) containing 0.1% Pluronic F68 (Thermo Fisher Scientific, Waltham, MA, USA) [17].

### 2.2. RNA Isolation, RT-PCR and Cloning of the Full-Length cDNA of BmADAR

Total RNA was extracted from the whole body, various tissues of the fifth instar larvae on day 3 or BmN cells using TRIzol reagent (Sangon, Shanghai, China) following the manufacturer’s instructions. Subsequently, DNase I (TaKaRa, Dalian, China) was used to treat the RNA to remove genomic DNA. RevertAid First Strand cDNA Synthesis Kit (Sangon, Shanghai, China) was used to synthesize cDNA according to the instructions. The primer pair of BmADAR-F and BmADAR-ER for amplifying a portion of the *BmADAR* fragment was designed based on the sequence of *BmADAR* (accession number, XM_004925094.1) registered in the silkworm genome database (http://silkbase.ab.a.u-tokyo.ac.jp/cgi-bin/index.cgi) [18] and the National Center for Biotechnology Information (NCBI) database. The total RNAs for BmADAR isoforms clonings were extracted from the whole bodies of three fifth instar larvae on day 3. Reverse transcription PCR (RT-PCR) was performed in the analysis of the gene transcriptional level in BmN cells or silkworm larvae. RT-PCR cycling conditions were 94 °C for 5 min, and then 35 cycles of 94 °C for 10 s, 55 °C for 20 s, and 72 °C for 2 min, followed a final elongation step at 72 °C for 10 min. The PCR products were cloned into pGEM^®^ -T easy vectors (Promega, Madison, WI, USA) and sequenced (Sangon, Shanghai, China).

cDNA ends of *BmADAR* were obtained by rapid amplification of cDNA end (RACE) assay using SMARTer^TM^ RACE cDNA Amplification Kit (Clontech, Shanghai, China). Based on the sequencing results of the amplified partial *BmADAR* gene, three specific primers were designed for 5′-RACE, and two specific primers were designed for 3′-RACE. Through sequencing multiple positive clones of intermediate fragments, 5′-end or 3′-end containing fragments, several full-length cDNA sequences of *BmADAR* were obtained. Then, primers for cloning the full-length of *BmADAR* cDNA were designed and RT-PCR was performed to isolate more different *BmADAR* transcripts. In addition, the expression patterns of different *BmADAR* transcripts were examined by RT-PCR with each specific primer pair. All of the primers used in this study are shown in Table S1.

### 2.3. Sequence Analysis

Genetyx_version 7 software was used to overlap cDNA sequences and analyze the ORF of each transcript. A translation tool on NCBI (https://www.ncbi.nlm.nih.gov/orffinder/) was used to translate the full-length cDNA of *BmADARa* into the protein sequence. The NCBI blast server was used to search the homologous sequences of ADAR. The phylogenetic tree was created using the MEGA version 3.1 Neighbor-Joining algorithm.

### 2.4. Overexpression of BmADARa and Preparation of the Polyclonal Antibody

In order to construct prokaryotic expression vector, the coding region of *BmADARa* with a 6xHis-tag at C-terminus was amplified by PCR and pMD19T was connected for sequencing verification. The primers used in PCR are shown in Table S1. After being digested with *Hind* III and *Nde* I, T4 ligase was used to connect PCR products to pET24b vector within 2 h at 16 °C. The 6xHis labeled prokaryotic expression plasmid was then transformed into *E. coli* Transetta (DE3) cells. The recombinant BmADARa was expressed and purified according to the methods of Rao et al. [19]. Approximately 3 mg purified recombinant protein was collected to prepare rabbit polyclonal anti-BmADARa antiserum [20,21].

### 2.5. Extraction of Total Proteins from Larval Tissue and Adenosine Deaminase Activity Assay

Crude proteins were extracted from each group of larval tissues using a One-Step Animal Tissue Active Protein Extraction Kit (Sangon, Shanghai, China) following the manufacturer’s instructions. Each sample contained three independent individuals to eliminate any individual differences. The activity of adenosine deaminase (ADA) was determined as previously described [22]. Total tissue proteins (100 µg) were added to the reaction mixture containing 50 mM sodium phosphate buffer at pH 7.0. The samples were preincubated for 10 min at 37 °C, and the reaction was started by the addition of substrate (adenosine) to a final concentration of 3 mM in a final volume of 200 µL. After 30 min, 500 µL phenol-nitroprusside reagent (50.4 mg of phenol and 0.4 mg of sodium nitroprusside/mL) was added to stop the reaction. ADA activity was determined by a spectrophotometric method using the Betelot reaction [23] at fixed intervals to measure the resulting ammonia, which contained a 500 µL of alkaline-hypochlorite reagent (0.6 M NaOH containing 0.125% sodium hypochlorite available chlorine). The samples were vortexed and incubated at 37 °C for 15 min, and then colorimetric analysis was performed at 635 nm. The enzyme activity was expressed in nmol NH_3_ min^−1^ mg^−1^ protein. Only adenosine was added to the control without protein extraction to correct the non-enzymatic hydrolysis of the substrate. All enzyme reactions took place in approximately five separate samples.

### 2.6. Construction of Recombinant Plasmids and Identification of Recombinant Bacmids

To construct the Bac-to-Bac/BmNPV expression system, PCR products of BmADARa ORF amplified from the full length cDNA were purified using a DNA purification kit (Promega, Madison, WI, USA), digested with *Nde* I and *Hind* III (TaKaRa, Dalian, China), cloned into the multiple cloning sites with polh promoter of the pFastBac^TM^ Dual vector and confirmed by sequencing (Invitrogen, Shanghai, China). The reprot gene GFP was cloned from pMD19T-GFP by primer pairs GFP F/R and inserted into the multi clone sites of pFastBac^TM^ Dual vector (*Xhol* I and *Kpn* I). The recombinant plasmid was named BmADARa-pFastBac^TM^ Dual, which was extracted and transformed into *E. coli* DH10Bac (BmNPV) cells. Positive recombinant bacmid was identified using RV-M and M13-47 primers [24].

Similarly, to overexpress *BmADARa* in insect cell lines, PCR products of BmADARa ORF amplified from the full length cDNA were purified and digested with *Sac* I and *Xba* I, then cloned to the pIZ/V5-His-EGFP vector. At the same time, according to the silkworm genome database (http://sgp.dna.affrc.go.jp/KAIKObase/), NCBI database and the information by Yang et al. [15] and Yin [25], a 1948-bp genomic fragment of BmSynaptotagmin I (BmSyt I, NM_001160200.1) was selected which contains the eighth exon, the eighth intron, and the first 92 bp of the ninth exon. PCR products of BmSyt I fragment were purified and digested with Hind III and BamH I and constructed to the pIZ/V5-His-EGFP vector. The recombinant plasmids were confirmed by sequencing and named BmADARa-pIZ-EGFP and BmSyt I-pIZ-EGFP, respectively.

### 2.7. Expression and Purification of Recombinant BmADARa in BmN Cells

The recombinant bacmid BmADARa-pFastBac^TM^ Dual was transfected into BmN cells according to the instructions of Lipofectamine2000 Reagent (Lifetechnology, Shanghai, China). Cell cultures and supernatant passage virus were collected, and the expression of recombinant BmADARa in BmN cells was performed according to the methods of Gan et al. [21]. After the remanent proteins had been eluted from the Ni-NTA agarose column, the protein was eluted and concentrated according to a previous study [24]. The purified protein was then stored in Tris-HCl buffer at pH 7.4, as described by Li et al. [26] and stored at 4 or −80 °C until use. One microgram of purified protein was used in each reaction in the ADA activity assay as described above.

### 2.8. Subcellular Localization of BmADARa

To observe the subcellular localization of BmADARa directly, Sf9 cells were removed from 75% ethanol using a coverslip and gently placed in a 6-well culture plate. Irradiation was carried out for 2–3 h at a distance of 20–30 cm from the direct range of the ultraviolet lamp, after which the cells were grown overnight on a glass sheet for confocal microscopy (Olympus, Tokyo, Japan). When 80% of the Sf9 cells converged, the cells were inoculated into 6-well cell culture cluster (Beaver) (2 × 10^5^ cells per well) for the transient transfection. The plate was incubated at 37 °C for 48 h in a 5% CO_2_ water bath incubator. When the adherent cells were grown to cover 2/3 of the bottom of the plate, the medium was removed and 2 µg of BmADARa-pIZ-EGFP plasmid was transfected into the corresponding well by an Effectene Transfection Reagent Kit (QIAGEN, Dusseldorf, Germany). The transfected cells were washed, fixed and stained according to the method of Yu et al. [27], and then photographed using a Nikon Eclipse TE 2000-E Confocal Microscope (Nikon, Japan). Each image shown is a representative example of n ≥ 5. Transfection of pIZ/V5-EGFP was used as a control.

### 2.9. Overexpression of BmSyt I and Co-Expression with BmADARa

After 80% of the BmN cells confluented, the cells were inoculated into a 6-well cell culture cluster (Beaver) for the transient transfection. When the cells 60–80% confluented, 2 µg of BmSyt I-pIZ-EGFP plasmid was transfected into the corresponding well using the same transfection reagent as above. In the co-expression experiment, BmADARa-pIZ-EGFP (2 µg) and BmSyt I-pIZ-EGFP (2 µg) were co-transfected into BmN cells. After transfection for 48 h, a fluorescence microscope (Olympus, Tokyo, Japan) was used to observe the transfection efficiency of the cells. The relative transcriptional level of *BmSyt I* was determined by quantitative real-time PCR (qRT-PCR). qRT-PCR of *BmSyt I* was conducted using SYBR Premix Ex Taq II (Tli RNaseH Plus) (TaKaRa, Dalian, China) according to the manufacturer’s instructions in a CFX96 system (Bio-Rad, Hercules, CA, USA). *Bmrp49* was used as an internal control. The cycling conditions were 95 °C for 10 min, then 40 cycles at 95 °C for 10 s, 60 °C for 15 s, 72 °C for 20 s. At the end of each qRT-PCR reaction, a melting curve was generated to confirm a single peak and the possibility of primer-dimer and non-specific product formation was ruled out. The 2^−ΔΔ*C*t^ method [28] was used to calculate the relative expression level of *BmSyt I.*

In addition, *BmSyt I* was cloned with Syt I-RT-F and Syt I-RT-R as upstream and downstream primers after transfection. RT-PCR amplicons were directly sequenced after gel purification and cloned into the pMD19-T vector (TaKaRa), then the pMD19-T-BmSyt I plasmid was transformed into *E. coli* DH5α competent cells, and the positive clones were random selected for sequencing analysis. The target editing site of *BmSyt I* (A1098) was sequenced from cDNA clones and compared with its genomic DNA sequence.

### 2.10. Western Blot

Total proteins were extracted from larval tissues as described above and from BmN cells using the Tissue or Cell Total Protein Extraction Kit (Sangon, Shanghai, China). The nucleoprotein and cytoprotein of Sf9 cells in the subcellular localization experiment were extracted using the Membrane, Nuclear and Cytoplasmic Protein Extraction kit (Sangon, Shanghai, China). Separation, transfer and immunoblot analysis of proteins was performed as described in previous reports [26,29]. The antibodies of LaminB1 and GAPDH were purchased from Proteintech (Wuhan, China) and AtaGenix (Wuhan, China) respectively. The anti-BmADARa or anti-HA tag (Abcam, Cambridge, MA, USA) rabbit polyclonal antibody was diluted to 1:500 or 1:2500 and in 5% (v/v) skim milk in PBST, and the second antibody of goat anti-rabbit IgG conjugated with HRP (Sangon, Shanghai, China) was diluted 1:5000 in the same blocking buffer. The final detection was performed by using Enhanced HRP-DAB Chromogenic Substrate Kit (TIANGEN, Beijing, China).

### 2.11. Statistical Analysis

The ADA activity data are indicated as the mean ± standard deviation (SD) and analyzed by a one-way analysis of variance followed by Tukey’s test. The data of relative mRNA expressional level are shown as mean ± standard error (SEM). Two-way ANOVA and Tukey’s test were used to determine the statistical significance of the difference. Differences were considered statistically significant when the *p* value was less than 0.05.

## 3. Results

### 3.1. Molecular Cloning of BmADAR

**Figure 1:**
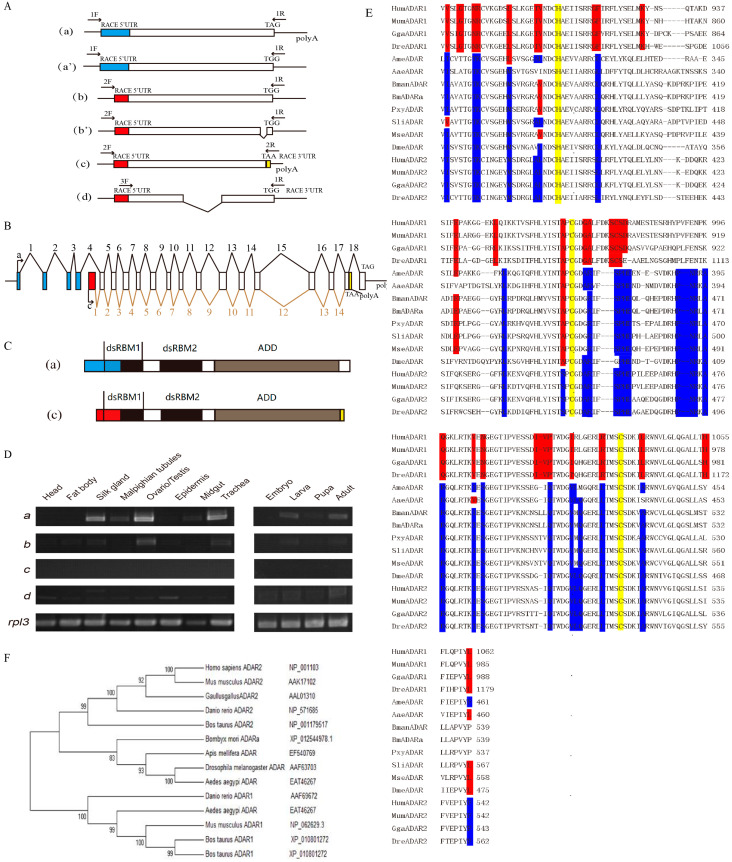
Molecular cloning and spatio-temporal expression of *BmADAR* transcripts and amino acid sequences analysis. (**A**) Different transcripts amplified from cDNA. Arrows indicate the location and direction of the primers. Straight lines are untranslated regions, white bars are coding sequences acquired by RT-PCR, while colored bars are coding sequences obtained by 5′-RACE and 3′-RACE. (**B**) Alternative splicing patterns and gene structures of transcripts a and c. Numbers indicate the exons. (**C**) Comparison of protein polypeptide structures between BmADARa and BmADARc. ADD, adenosine deaminase domain, dsRBM, double-stranded RNA binding motif. (**D**) Expressional patterns of BmADAR transcripts in different tissues from day 3 fifth instar larvae (left) and at different developmental stages (right, larval stage is the fifth instar). Transcription of *Bmrpl3* was used as a control. (**E**) Alignment of the adenosine deaminase domains among BmADARa and some known ADAR, ADAR1 and ADAR2 in insects and vertebrates. Red and blue represent the positions that were conserved in vertebrate *ADAR1* and *ADAR2*, respectively. Yellow indicates the chelated zinc residues. GenBank accession numbers are as follows: BmADARa, *Bombyx mori* ADARa, QJD20810; HumADAR1, *Homo sapiens* ADAR1, NP_001102; HumADAR2, *H. sapiens* ADAR2, NP_001103; MumADAR1, *Mus musculus* ADAR1, NP_062629.3; MumADAR2, *M. musculus* ADAR2, AAK17102; GgaADAR1, *Gaullus gallus* ADAR1, XP_001232162; GgaADAR2, *G. gallus* ADAR2, AAL01310; DreADAR1, *Danio rerio* ADAR1, AAF69672; DreADAR2, *D. rerio* ADAR2, NP_571685; AaeADAR, *Aedes aegypi* ADAR, EAT46267; DmeADAR, *Drosophila melanogaster* ADAR, AAF63703; AmeADAR, *Apis mellifera* ADAR, EF540769; BmanADAR, *Bombyx mandarina* ADAR, XP_028037491; MseADAR, *Manduca sexta* ADAR, XP_030029950; SliADAR, *Spodoptera litura* ADAR, XP_022835289 and PxyADAR, predicted *Plutella xylostella* ADAR, XP_011557396. (**F**) The neighbor-joining tree of ADARs was obtained by bootstrap analysis with the option of heuristic search. The bars indicate the distance of 1000 assessments of the bootstrap test confidence level.

A 1900 bp cDNA fragment was amplified by using RT-PCR with primers designed according to the sequences of *Bombyx mori ADAR* (Gene ID: 100101209). Then, 5′- and 3′-termini of *BmADAR* cDNAs were obtained by RACE. Nucleotide sequences splicing and analysis revealed there are six theoretical transcripts, four with complete ORFs and two with only start codons without stop codons. In fact, two transcripts with complete ORF (Figure 1A(a,c)), three transcripts without a stop codon (Figure 1A(a’,b,b’)) and a selective splicing of BmADARb (Figure 1A(d)) were successfully obtained by RT-PCR. Comparing the information in the silkworm genome database, *BmADARa* consists of 19 exons and 18 introns. The transcript c is a selective splicing product of *BmADARa*. For the *BmADARa* isoform, the length of the cloned 5′/3′ UTRs was 44 and 55 bp, respectively. Gene structure of *BmADARc* includes 15 exons (the coding region starts from the middle of the 4th intron of *BmADARa*) and 14 introns (corresponding to introns 4–17 of *BmADARa*, respectively) (Figure 1B). Moreover, there are terminal differences at both the amino- and carboxy-terminus between BmADARa and BmADARc, resulting in a partial amino acid difference in dsRBM1 (Figure 1C).

### 3.2. Expression Pattern of BmADAR Transcripts and Sequence Analysis of BmADARa

We analyzed the expression patterns of each *BmADAR* transcript by RT-PCR in different larval tissues and four developmental stages. Transcript a (including a and a’) was highly expressed in the larva and adult, especially in the larval silk gland, gonads and trachea. The expression pattern of transcript b (including b and b’) was similar to that of a, but the overall expression level was lower than for a (Figure 1D). Using sequencing analysis, we found that the single band was transcript b, but no transcript b’ was found, probably because the transcript b’ was expressed at a very low amount. The transcript c (Genbank: MN267833) was hardly expressed in any tissue or developmental stage (Figure 1D). In addition, we stumbled upon a small transcript d with low expression in different tissues and developmental stages (Figure 1D) when using BmADAR-F3 and BmADAR-R1 as RT-PCR primers (Figure 1A). Sequencing results indicated that transcripts d was another selective splicing product of b (Figure 1A).

The ORF length of *BmADARa* is 2154 bp, encoding 717 amino acids with a predicted molecular weight of 78.7 kDa and a theoretical isoelectric point of 9.04. Analysis of amino acid sequences showed that BmADARa has two dsRBMs and an adenosine deaminase domain (Figure 1C), which is predicted to be a double-strand-editing enzyme. In addition, BmADARa was compared with the deaminase domains of ADAR1 and ADAR2 in other species, and it was found that the three zinc chelation critical sites (His and Cys) of the catalytic center were highly conserved in all species (Figure 1E). Based on the conserved amino acid position of the deaminase domain of *ADAR1* or *ADAR2*, the insect *ADAR* gene is highly homologous to the vertebrate *ADAR2* (Figure 1E). The evolutionary analysis of the genetic relationships of amino acid sequences also leads to the same conclusion (Figure 1F).

### 3.3. Over-Expression of BmADARa and Adenosine Deaminase Activity

**Figure 2:**
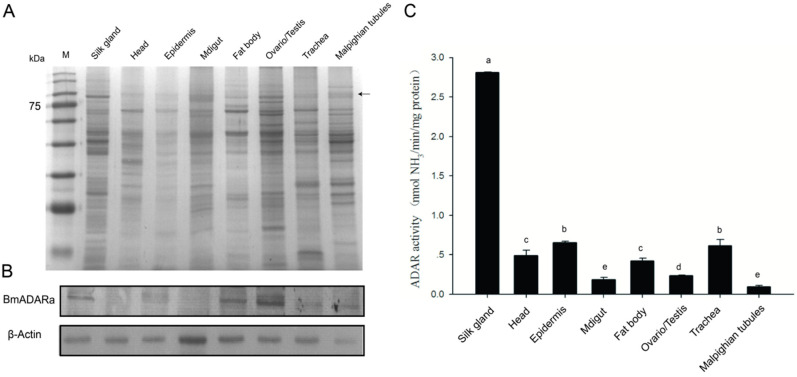
Identification of BmADARa expression and adenosine deaminase activity in the silkworm larval tissues. Ten milligrams of each tissue protein from day 3 fifth instar larvae were analyzed by SDS-PAGE (**A**) and Western blot (**B**) using anti-BmADARa antiserum. Arrow at the right shows the approximate position of BmADARa. BmActin was used as the control. Vertical bars indicate the mean ± SEM (n = 3). In addition, adenosine deaminase activity in 10 mg of total tissue proteins was determined (**C**). Bars represent the mean ± SD of at least five independent experiments performed in triplicate. Significance difference is shown between two alphabets (one-way ANOVA, followed by Tukey’s test as post hoc). The specific enzyme activity is shown as nmol of NH_3_ min^−1^mg^−1^ of protein.

**Figure 3:**
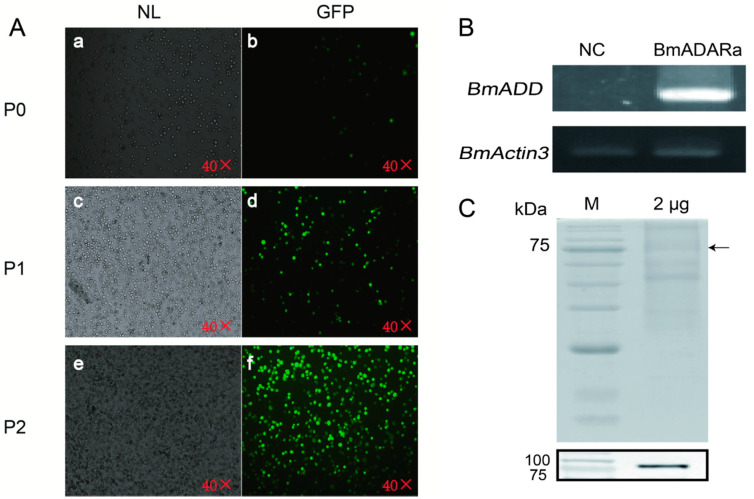
Over-expression and purification of BmADARa in the BmN cells by immunofluorescence (**A**), RT-PCR (**B**), SDS-PAGE and Western blot (**C**). P0, passage 0 virus; P1, passage 1 virus; P2, passage 2 virus. NL, under the normal light; GFP, under the green fluorescence microscope. Transcription of *BmActin3* gene was used as a positive control. NC, negative control. Arrow at the right shows the approximate position of BmADARa.

To prepare an anti-BmADARa polyclonal antibody, recombinant BmADARa was expressed and purified (Figure S1). SDS-PAGE was used to analyze 10 mg of each tissue protein of day 3 fifth instar larvae (Figure 2A). Western blotting was performed to identify the expression of BmADARa in the total tissue proteins. The results show that BmADARa was mostly expressed in the gonads, fat bodies and silk gland (Figure 2B). Adenosine deaminase (ADA) activity assay showed that the enzymatic activity from the silk gland was the highest, reaching 2.812 nmol of NH_3_ min^−1^ mg^−1^/mg protein (U) (Figure 2C). Together with the consistently high expressional level of BmADARa in the silk gland (Figure 1D, Figure 2B,C), BmADARa could play an important role in the A-to-I RNA editing for genes expressed in larval silk gland. Thereafter, BmADARa was co-expressed with GFP in BmN cells through the Bac-to-Bac/BmNPV expression system (Figure 3A). Over-expression of *BmADARa* was identified by RT-PCR (Figure 3B). SDS-PAGE and Western blot analysis revealed that BmADARa existed in the intracellular space (Figure 3C). ADA activity of the purified recombinant protein was 0.184 U, obviously lower than that in the silk gland (Figure 2C).

### 3.4. Subcellular Localization of BmADARa and Its Editing Function on BmSyt I

**Figure 4:**
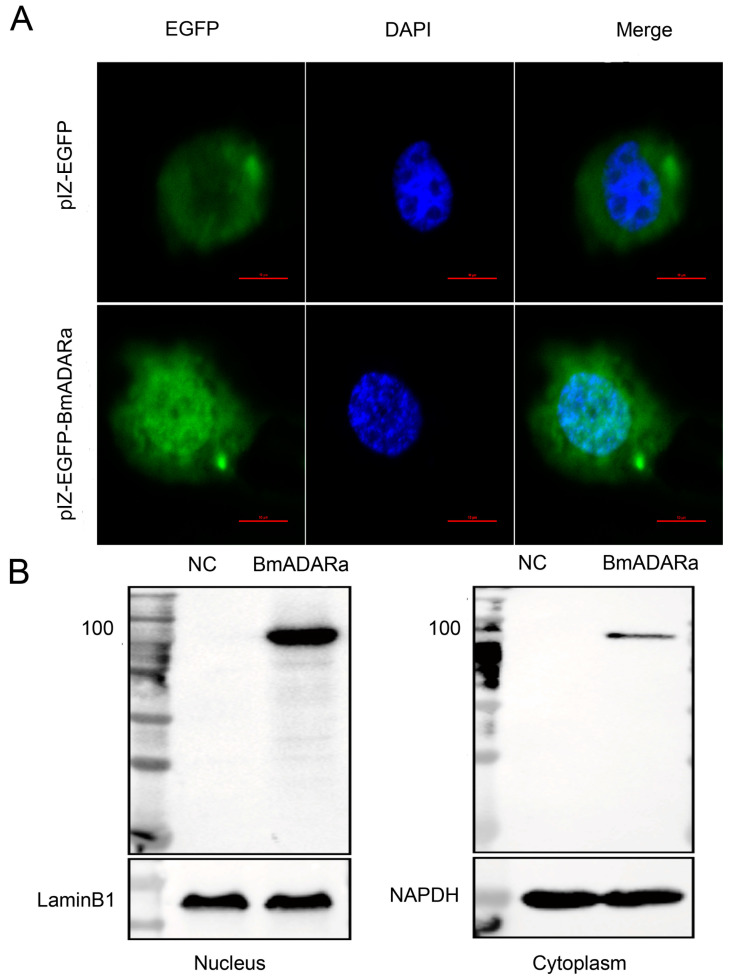
Subcellular localization of BmADARa in the Sf9 cells (3000×). (**A**) BmADARa co-localizes with DAPI-stained nuclei. pIZ/V5-EGFP was used as a control. Scale bar, 10 µm. (**B**) Western blot analysis of the expression of BmADARa in the nucleus and cytoplasm using anti-BmADARa antibody. LaminB1 and GAPDH were used as the respective controls. NC, negative control.

In order to study the subcellular localization of BmADARa in cells, we constructed a BmADARa-pIZ-EGFP expression vector. After transfection into Sf9 cells for 48 h, green fluorescence is observed in both the nucleus and the cytoplasm, and BmADARa is mainly distributed in the nucleus with a little in the cytoplasm (Figure 4A), while the control cells transfected with pIZ/V5-His-EGFP showed more green fluorescence in the cytoplasm (Figure 4A). The Western blot assay also confirmed that the BmADARa protein was located in both the nucleus and cytoplasm, but mainly in the nucleus (Figure 4B), supporting that the enzyme has an editing function on the substrate dsRNA in the nucleus.

**Figure 5:**
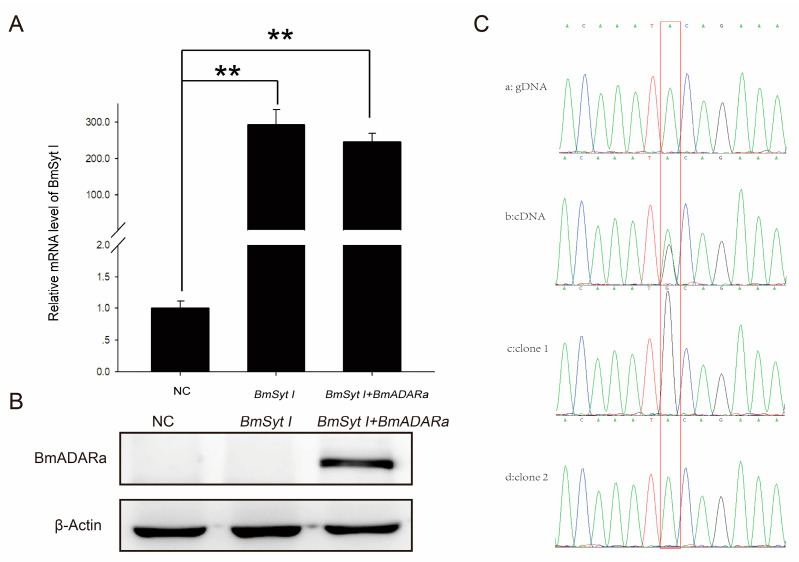
Identification of the A-to-I RNA editing function of BmADARa on *BmSyt I* in the BmN cells. (**A**) qRT-PCR analysis of *BmSyt I* transcriptional level after BmSyt I-pIZ-EGFP transfection or co-transfected with BmADARa-pIZ-EGFP. *Bmrp49* was used as an internal control. Bars represent the mean ± SEM (n = 3) of at least three independent experiments performed in triplicate. The asterisk represents a significant difference (two-way ANOVA, followed by Tukey’s test as post hoc, ** *p* ≤ 0.01). (**B**) Western blot analysis after BmSyt I-pIZ-EGFP transfection or co-transfected with BmADARa-pIZ-EGFP using anti-BmADARa or anti-actin antiserum (positive control). NC, negative control. (**C**) Sequence comparison of *BmSyt I* among genomic DNA, cDNA and recombinant clones. a, direct sequencing map of genomic DNA PCR product of *BmSyt I*; b, direct sequencing map of RT-PCR product of *BmSyt I* in the BmADARa/BmSyt I co-transfected cells. c, sequencing map of a single clone of A-to-I RNA edited *BmSyt I* from RT- PCR product in the *BmADARa*/*BmSyt I* co-transfected cells. d, sequencing map of a single clone of A-to-I RNA unedited *BmSyt I* from RT- PCR product in the *BmADARa*/*BmSyt I* co-transfected cells. Red bar indicates A/G editing site.

Moreover, the BmSyt I-pIZ-EGFP was transfected into BmN cells alone or co-transfected with BmADARa-pIZ-EGFP expression vector. After transfection for 48 h, over-expression of *BmSyt I* in both cases was observed by qRT-PCR (Figure 5A). In the case of two vectors co-transfection, expression of BmADARa was confirmed by Western blot analysis (Figure 5B). By direct sequencing and comparing between genomic PCR product of *BmSyt I* and the RT-PCR product of *BmSyt I* after co-transfection with *BmADARa* (MN398636), a distinct A/G doublet was found at the point (Figure 5C(a,b)), suggesting that the BmADARa-induced A-to-I editing event happened there. Subsequently, by cloning and sequencing, two kinds of cDNA clones with A or G at this editing site were isolated (Figure 5C(c,d)), further verifying that BmADARa has an A-to-I RNA editing function for a specific site of *BmSyt I* (A1098G).

## 4. Discussion

RNA editing refers to the fact that the mRNA produced by gene transcription is inconsistent with the gene coding sequence due to the deletion, insertion or substitution of nucleotides during transcription, and even the encoded amino acid sequence of the translation and the encoded information in the gene sequence may appear to be different [6,30]. Here, six transcripts encoding different amino and carboxyl termini were obtained at Dazao by RT-PCR and RACE, including two transcripts with complete ORF sequences, and three no stop codons and a selective splicing of *BmADARa* (Figure 1A). These six transcripts are formed by the selective splicing and RNA editing of BmADAR mRNA precursors during transcription.

Sequence alignment between BmADARa and a double-stranded RNA-specific editase ADAR isoform X1 (XM_012689524) predicted by *B. mori* genome project (PRJNA205630) showed that they share 99.95% nucleotides identity in CDSs, and the partial 5′/3′ UTRs of BmADARa obtained in this study are 100% identical to that of ADAR X1. Therefore, we think that *BmADARa* is the same transcript as ADAR X1. In addition, BmADARc isoform resembles the transcript variants ADAR X2 (XM_012689525), ADAR X3 (XM_012689526) and ADAR X4 (XM_021347298). Interestingly, these silkworm ADAR isoforms appear well conserved between *B. mori* and its original ancestor *Bombyx mandarina* (genome project accession: PRJNA522300), supported by the fact that BmADARa and BmADARc are similar to *B. mandarina* ADAR X1 (XM_028181690) and *B. mandarina* ADAR X2 (XM_028181691), respectively. Moreover, comparative genomic analyses showed that the Lepidoptera insect ADARs are highly conserved among *B. mori*, *B. mandarina*, *Manduca sexta*, *Spodoptera litura*, and *Plutella xylostella* (Figure 1E). Further homologous sequence alignment and conservation analysis indicated that *BmADARa,* as for other insect ADARs such as in *D. melanogasterr* [31], belongs to the homolog family of vertebrate ADAR2 (Figure 1E,F). As *Drosophila* ADAR and p110/p150 isoforms in vertebrate ADAR1 have different transcription start sites [3,32], *BmADAR* is rich in transcripts in the silkworm, and its structure is complex (Figure 1A) [33]. The difference is that the expression of *Drosophila* ADAR transcripts is development-specific; that is, some transcripts are only expressed during a specific developmental period [34], and the temporal and spatial expression patterns of *BmADAR* transcripts are not identical—rather, at a particular developmental stage, each transcript appears to be expressed or not (Figure 1D). The spatiotemporal expression pattern showed that the silkworm mainly expressed the *BmADARa* subtype (Figure 1D).

ADA hydrolyzes adenosine as a neuromodulator to inosine in a manner similar to ADAR catalyzing the water-free amino reaction on dsRNA substrates [35]. Some reports have shown that ADA plays a very important role in the transmission of adenosine signals from the central nervous system, the immune response to inflammation and the diagnosis of diseases in the humans and *Drosophila melanogaster* [22,36,37]. However, there are few reports on the ADA activity of ADAR. In this study, we examined ADA activity of both purified recombinant BmADARa and several larval tissue proteins. Our data showed that BmADARa held ADA activity in vitro, although the total proteins in silkworm silk gland displayed significant highest hydrolysis ability of adenosine (Figure 2C). The lower ADA activity of purified BmADARa may be due to the poor preservation of enzyme activity during the purification or in the assay conditions. We are convinced that alternative techniques exist to evaluate the exogenous expression and purification of ADARs in vitro and have made meaningful attempts. Considering that end-point RT-PCR and Western blot are not quantitative techniques, some factors may affect the stability of mRNA and protein as well as enzyme activity, so that inconsistency between the expression profiles (Figure 1D), Western blot (Figure 2B) and ADA activity (Figure 2C) of certain tissues, such as fat body and gonads occurred. This also reflects that the change in editing level is usually not related to the change in mRNA expression of editing enzyme [38]. The subcellular localization of ADAR proteins is crucial to the regulation of its editing efficiency. Most of the RNA-editing events occur in the nucleus, which is where the RNA substrates of ADARs are primarily localized [9]. Vertebrate ADAR2 is generally localized in the nucleus, and analyses indicate that this is due to the presence of a nuclear localization signal (NLS) [4,38,39]. However, the human ADAR1 carries a unique NLS that overlaps one of its dsRBMs, and this dsRBM-NLS is recognized by the nuclear import receptor transportin 1 (Trn1) [40], while the integrity of dsRBM1 and dsRBM2 domains are necessary for proper nuclear localization of ADAR2 [41]. In this study, BmADARa was mainly localized in the nucleus, with only a small amount of expression in the cytoplasm (Figure 4), supporting the hypothesis that BmADARa mainly plays an important role in the A-to-I RNA-editing events in the nucleus. In addition, as a speculative hypothesis, we can consider the preferential localization in the cytoplasm of the BmADARc isoform, in which a partial deletion of the dsRMB1 domain occurs (Figure 1C). The potential cytoplasmic substrate for RNA editing can be viral RNA, which can induce proviral and antiviral effects, or retain the mRNA at the editing site after processing [42], while ADAR1 plays an important role in innate immunity and virus replication [43]. Together with especial expression in the larval silk gland, gonads and trachea (Figure 1D), we think that BmADARa performs RNA-editing function actively in these tissues. The relationship between the expression of BmADARa in the cytoplasm and viral RNA remains to be further studied.

ADARs are capable of specifically recognizing adenine sites on dsRNA substrates and are also capable of completely deactivating multiple adenine sites for deamination, both of which are determined entirely by the secondary structure of the edited RNA [44]. More than 20 bps of dsRNA structures formed intramolecularly or intermolecularly are likely to be substrates for ADARs [45]. In addition, there are very few genes (e.g., *4f-rmp* in *Drosophila* and *eri-6* in nematodes) that can form complementary RNA pairing sequences that are affected by RNA editing [31,46]. By comparing the sequencing map of BmN cell cDNA amplification products after co-transfection of *BmADARa* and *BmSyt I*, one can judge whether an editing event occurs, and this is a straightforward and rapid way to find the A-to-I editing site of the target gene (Figure 5C).

An imperfect folded-back dsRNA structure is formed between the sub-complementary sequences as a substrate for ADARs. For example, glutamate receptor B (GluR-B) Q/R site of pre-mRNA [33], GluR-B, -C, -D R/G site [47], serotonin (5-HT) A/E site of receptor 2C (5-HT_2C_R) [48]. In addition, dsRNA structures can form editing-site complementary sequences (ECS) through complex long-range pseudo-binding, such as in several re-encoding sites identified in *Drosophila synaptotagmin I* [11]. In summary, ECS and dsRNA structures are essential for RNA editing of A-to-I. When *Drosophila* ADAR gene was co-transfected with *BmSyt I* to S2 cells, A1098G (a putative editing site exists in the 8th exon of *BmSyt I*) transition took place, in which the more than 1000 bp 8th intron was used as ECS [25]. In this study, we firstly predicted the BmADARa-mediated pre-mRNA structure of *BmSyt I* (http://rna.tbi.univie.ac.at/cgi-bin/RNAWebSuite/RNAfold.cgi), and we found that a dsRNA-like stem-loop structure appeared at the A-to-I RNA-editing site of A1098 (data not shown). Then, by co-transfection of *BmADARa* and *BmSyt I* to BmN cells, the same A1098G edition was observed (Figure 5C), proving that such a ECS containing structure can surely guide BmADARa to recognize specific editing site of *BmSyt I* and act on there.

A variety of in vitro and mutant experimental evidence indicates that ADARs recognize the specific A site and deaminate through the secondary structure formed by the dsRNA substrate, and the precise location of the editing site in the substrate and the degree of editing are determined by the substrate itself [22,37,39]. Although the secondary structure is determined, until now it has not been possible to predict which A is the target site for ADAR editing based on the secondary structure of RNA. By studying the cloning, expression pattern, subcellular localization and A-to-I RNA-editing *BmSyt I* of BmADARa, the results of this report indicate that BmADARa mainly exists in the nucleus (Figure 4) and has editing function for a specific site of *BmSyt I* (Figure 5C). Next, we will analyze the function of predictive NLS and each domain of BmADARa to know how NLS mediates its entry into the nucleus and the recognition and action mode of the substrates, hoping to further reveal the molecular regulation mechanism of BmADARa-mediated A-to-I RNA-editing processes in the silkworm *B. mori*.

## 5. Conclusions

The deamination of adenosine into inosine (A-to-I) is catalyzed by the adenosine deaminase acting on the RNA (ADAR) protein family. In this study, six ADAR transcripts encoding different amino and carboxyl termini were obtained in the silkworm, *Bombyx mori* (BmADAR), which are formed by selective splicing and RNA editing of BmADAR mRNA precursors during transcription. *BmADARa* is a mainly expressed transcript with complete ORF sequence length of 2154 bp, encoding 717 amino acids with two dsRBMs and an adenosine deaminase domain, highly homologous to the vertebrate ADAR2. BmADARa mainly exists in the nucleus and has editing function for a specific site of *BmSyt I*. This study provides insight into the function of the first ADAR enzyme in *B. mori*, also lays the foundations for further exploration of the functional domain of BmADARa and its editing substrates and target sites.

## Supplementary Materials

The followings are available online at https://www.mdpi.com/2075-4450/11/8/523/s1. Figure S1: Expression and identification of recombinant BmADADa in the *E. coli* transetta (DE3) strain, Table S1: Primer sequences used in this study.
