# Supplementary material for: Expressional Localization and Functionally Identifying an RNA Editing Enzyme BmADARa of the Silkworm Bombyx mori
Source: Insects. 2020 Aug 12;11(8):523. doi: 10.3390/insects11080523 (PMC7469206; doi:10.3390/insects11080523)
Supplement: Supplementary file 1 [file insects-11-00523-s001.zip › Supplementry/Table S1.docx]

Table 1 Primer sequences used in this study

| **Assay** | **Primer sequence (5’ to 3’)** | **Primer name** |
| --- | --- | --- |
| RT-PCR | BmADAR-F | AGCGCATTGTCCCAGCTTCA |
|  | BmADAR-ER | TCATCACCTCGGGCTGTATCGT |
|  | BmADAR-F1 | ATTACAATAGAACGCTTAAAGAGTCC |
|  | BmADAR-F2 | GACAACAGACAAAATGCCAAAT |
|  | BmADAR-F3 | CGGTCTTTGGAGTTGCTATG |
|  | BmADAR-R1 | GCCGGGTCAGCTTAGTATATAT |
|  | BmADAR-R2 | GGATTTCCCGAGGTTTACTTTC |
|  | BmADARa-F | ATTACAATAGAACGCTTAAAGAGTCC |
|  | BmADARa-R | GCCGGGTCAGCTTAGTATATAT |
|  | rpl3-F | TCGTCATCGTGGTAAGGTCAA |
|  | rpl3-R | TTTGTATCCTTTGCCCTTGGT |
|  | BmADD-RT-F | CCGACATCGCGGACA |
|  | BmADD-RT-F | ACAGAGCGGCTGGGTC |
|  | Actin3-F | AGACGAGGCACAGAGCAA |
|  | Actin3-R | TGTAGAAGGTATGATGCCAAA |
|  | Syt I-RT-F | ATCCGTACGTAAAGATTGC |
|  | Syt I-RT-R | GACGCGTTGTAACCAA |
|  | RV-M | GAGCGGATAACAATTTCACACAGG |
|  | M13-47 | CGCCAGGGTTTTCCCAGTCACGAC |
| RACE | BmADAR 5’ GSP1 | GGAGACGGGATAAGGTGTTGT |
|  | BmADAR 5’ GSP2 | CCGAACAGCGACGACGTGGGCAGA |
|  | BmADAR 5’ GSP3 | ATCTGGCAGACAACCTTGAACCTC |
|  | BmADAR 3’ GSP1 | GCGAGCGCCTCCTGACGATGTC |
|  | BmADAR 3’ GSP2 | TATGCGGGCGCATCGAAGCGTACAT |
| qRT-PCR | Syt I-qRT-F | GGACAACAAGCTGGGAGACAT |
|  | Syt I-qRT-R | CGTACGGATCTGAGAGACCAC |
|  | rp49-qRT-F | CCCAACATTGGTTACGGTTC |
|  | rp49-qRT-R | GCTCTTTCCACGATCAGCTT |
| Expression  vectors construction | ADAR-24b-F | GGAATTCCATATGCATCATCATCATCATCATTATAAGCCAATAGAT |
|  | ADAR-24b-R | CCCAAGCTTAACTAGATCTGTCTAAGTGAACAGAGCGGC |
|  | *GFP* F | CCGCTCGAGATGGTGAGCAAGGGC |
|  | *GFP* R | CGGGGTACCTTACTTGTACAGCTC |
|  | ADAR-pFastDual-F | CGCGGATCCATGTATAAGCCAATAGATTTC |
|  | ADAR-pFastDual-R | CCCAAGCTTCTAATGATGATGATGATGATGAGTGAACAGAGCGGCT |
|  | ADAR-pIZ-EGFP-F | TCCGAGCTCATGTATAAGCCAATA |
|  | ADAR-pIZ-EGFP-R | GCTCTAGACTAGTGAACAGA |
|  | Syt I-pIZ-EGFP-F | CCCAAGCTTATCCGTACGTAAA |
|  | Syt I-pIZ-EGFP-R | CGGGATCCGACGCGTTGTA |

Underlined nucleotides indicate restriction sites.
