# Supplementary figures and images for: Expressional Localization and Functionally Identifying an RNA Editing Enzyme BmADARa of the Silkworm Bombyx mori
Source: Insects. 2020 Aug 12;11(8):523. doi: 10.3390/insects11080523 (PMC7469206; doi:10.3390/insects11080523)

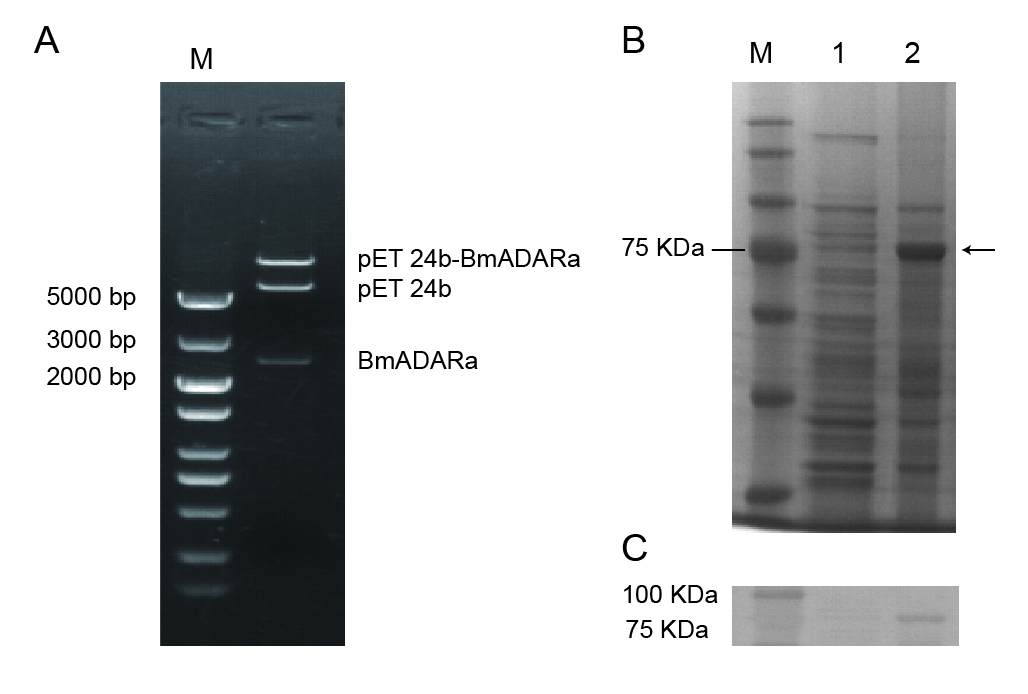

Supplement: Supplementary file 1 [file insects-11-00523-s001.zip › Supplementry/Figure S1.tif]
